# Association of 6:2 Fluorotelomer Ethoxylate Exposure with Serum Lipids in General Adults

**DOI:** 10.3390/toxics13080664

**Published:** 2025-08-07

**Authors:** Yan Wu, Qianjin Li, Rendi Deng, Rui Wang, Junfen Fu, Fangfang Ren, Hangbiao Jin

**Affiliations:** 1 Department of Endocrinology, Children’s Hospital, Zhejiang University School of Medicine, National Clinical Research Center for Child Health, Hangzhou 310014, China; 2 Department of Endocrinology and Metabolic Diseases, Zhejiang Greentown Cardiovascular Hospital, Hangzhou 310014, China; 3 Laboratory Department, Zhejiang Greentown Cardiovascular Hospital, Hangzhou 310014, China; 4 Key Laboratory of Microbial Technology for Industrial Pollution Control of Zhejiang Province, College of Environment, Zhejiang University of Technology, Hangzhou 310014, China

**Keywords:** 6:2 fluorotelomer ethoxylates, human serum, gender, age, lipid metabolism

## Abstract

A series of 6:2 fluorotelomer ethoxylates (FTEOs) has been recently detected in human serum. Whether it has the potential to disrupt lipid metabolism in human populations remains largely unexplored. This study quantified serum concentrations of 6:2 FTEOs in 237 healthy Chinese adults, examined the gender- and age-specific differences in serum levels of 6:2 FTEOs, and investigated the associations between serum levels of 6:2 FTEOs and lipid profiles for the first time. Nine 6:2 FTEO homologues were detected in collected human serum, with detection frequencies of 22–81%. 6:2 FTEO8 and 6:2 FTEO9 were the more abundant 6:2 FTEO homologues in human serum, displaying the mean levels of 0.69 ng/mL (range < LOD–7.36 ng/mL) and 0.71 ng/mL (<LOD–8.12 ng/mL), respectively. Male participants had much higher (*p* < 0.05) mean serum levels of 6:2 FTEO6 (0.61 vs. 0.31 ng/mL), 6:2 FTEO7 (0.44 vs. 0.21 ng/mL), 6:2 FTEO8 (0.91 vs. 0.38 ng/mL), and 6:2 FTEO11 (0.35 vs. 0.18 ng/mL) than female subjects. Correlation analysis revealed a significantly positive relationship (*p* < 0.01) between the age of participants and human serum concentrations of 6:2 FTEO6–6:2 FTEO11. Multivariate linear regression identified significant positive associations between specific 6:2 FTEO homologues (e.g., 6:2 FTEO6, 6:2 FTEO8–6:2 FTEO10) and elevated total cholesterol, high-density lipoprotein cholesterol, low-density lipoprotein cholesterol, and triglyceride levels.

## 1. Introduction

Poly- and perfluoroalkyl substances (PFASs) have been widely employed in industrial and consumer products due to their exceptional resistance to oils, water, and chemical degradation [1,2]. These properties have resulted in their pervasive presence in environmental matrixes and human blood worldwide [3,4,5]. Fluorotelomer ethoxylates (FTEOs) have recently emerged as preferred anti-fogging additives used in optical equipment like prescription lenses, protective visors, and imaging devices [6]. The COVID-19 pandemic has significantly accelerated their adoption in a lot of healthcare equipment (e.g., medical goggles and face shields), through application methods ranging from aerosol sprays to impregnated cleaning cloths [7]. This surge aligned with heightened demands for fog-resistant personal protective equipment during global health emergency responses [8,9].

Elevated aqueous solubility and low volatility enhance the mobility of FTEOs in ecosystems following their applications to treated surfaces. Emerging research has detected these compounds in wastewater effluents, consumer anti-fogging products, household dust, and human blood, with 6:2 FTEOs consistently emerging as the most prevalent homologues [10,11]. Structurally characterized by a six-carbon fluorotelomer backbone modified with ethoxylate substituents, 6:2 FTEOs exhibit environmental bio-transformation pathways that could generate environmentally stable PFASs [12]. This underscores the necessity for comprehensive risk assessments to evaluate human FTEO exposure to 6:2 FTEOs in human health frameworks.

Lipids, encompassing diverse biomolecules such as fatty acids, glycerolipids, and sterols, are essential for sustaining vital physiological processes in humans [13,14]. However, the dysregulation of lipid metabolism, such as elevated low-density lipoprotein cholesterol (LDL-C) and total cholesterol (TC), has been identified as an important driver of atherosclerotic cardiovascular disease [15,16,17]. Epidemiological evidence further linked increased serum triglyceride (TG) level concentrations to heightened cardiovascular disease risk in humans [18,19]. Epidemiological evidence suggests that PFASs, such as perfluorooctanoic acid (PFOA) and perfluorooctanesulfonic acid (PFOS), may dysregulate lipid homeostasis through diverse pathophysiological pathways, which include interference with endocrine signaling, the perturbation of nuclear receptors, and interactions with constitutive androstane receptor-mediated metabolic cascades [20,21]. Multiple population-based studies have demonstrated significant associations between PFAS exposure and altered serum lipid profiles, notably elevated total cholesterol (TC), triglycerides (TG), and low-density lipoprotein cholesterol (LDL-C) [22,23,24]. For example, dose-dependent associations had been reported for PFOA and PFOS with significant increases in serum levels of high-density lipoprotein cholesterol (HDL-C) [25,26]. The structural homology shared between 6:2 FTEOs and PFOA or PFOS raises concerns about their potential to similarly disrupt lipid regulatory networks in humans. Until now, the effects of human exposure to 6:2 FTEOs on lipid metabolism have not been well investigated.

In this study, we hypothesized that exposure to 6:2 FTEOs may disrupt the lipid regulatory networks in humans. To verify this hypothesis, we quantified the concentrations of 17 kinds of 6:2 FTEO homologues in serum samples from 237 healthy adults from Hangzhou City, China. In addition, concentrations of TC, TG, LDL-C, and HDL-C were also determined in collected human serum samples. Finally, we examined, for the first time, the potential associations between measured serum concentrations of 6:2 FTEO homologues and serum lipid profiles (TC, TG, LDL-C, and HDL-C) in human subjects. This study contributes to the better understanding of these emerging contaminants’ potential metabolic effects on humans and informing both public health risk assessment and chemical regulation priorities.

## 2. Materials and Methods

### 2.1. Chemical Standards and Reagents

The high-purity chemical standard (≥97%) of 6:2 FTEO8 was custom-synthesized and supplied by Jufu Technology (Hangzhou, China). The naming convention for 6:2 FTEO homologues followed previously reported guidelines [6,10]. A commercial 6:2 FTEO mixture (brand name FS-3100), which contained homologues ranging from 6:2 FTEO2 to 6:2 FTEO18, was obtained from Fuhong Chemical (Suzhou, China). The manufacturer provided detailed compositional data for each 6:2 FTEO homologue, as determined by fluorine-19 nuclear magnetic resonance (^19^ F NMR) analysis and shown in the supporting information (Supplementary Materials, Table S1). Isotopically labeled standards, including benzyldimethyldodecylammonium chloride-*d*_5_ and benzyldimethyltetradecylammonium chloride-*d*_7_, were purchased from Anpu Technology (Shanghai, China). The chemical structures of 6:2 FTEO homologues are shown in the Supplementary Materials, Figure S1. In addition, pure water, tetrabutylammonium hydrogen sulfate (TBAH), methyl tert-butyl ether (MTBE), ammonium acetate, methanol, acetonitrile, isopropanol, and acetic acid were purchased from Merck (Shanghai, China) and Fisher (New York, NY, USA).

### 2.2. Subjects and Human Serum Collection

From January to March 2025, healthy Chinese adults attending routine physical examinations at Greentown Cardiovascular Disease Hospital were recruited as study participants. The physical examination department of the Greenwood Cardiovascular Disease Hospital is specifically responsible for routine health check-ups for the general public. The study cohort consisted of 115 male and 122 female volunteers, who were randomly selected from the eligible population. Before enrollment, all participants provided written informed consent in accordance with ethical guidelines. The research protocol was reviewed and approved by the Human Ethics Committee of Greentown Cardiovascular Disease Hospital. Trained nursing staff conducted structured face-to-face interviews to acquire detailed demographic information from each participant.

Fasting venous blood samples (5 mL) were collected from individual subjects in heparinized BD Vacutainer tubes (Becton, Dickinson and Company, New Jersey, NJ, USA) by qualified nurses. After a 15-min resting period post-collection, collected blood samples were centrifuged at 3800× *g* to isolate the serum fraction, which was then aliquoted and stored at −80 °C until further analysis. Field blanks (*n* = 5) containing 5 mL of pure water were processed in parallel with the serum samples under the same conditions.

### 2.3. Analysis of 6:2 FTEOs in Human Serum

Extraction of 6:2 FTEOs from human serum was conducted based on previously reported techniques [10]. Briefly, 1.0 mL of human serum was fortified with internal standards (5.0 ng each) and subsequently mixed with 1 mL of 0.5 mol/L TBAH solution (pH adjusted to 10) and 2 mL of 0.25 mol/L sodium carbonate buffer. After thorough vortex mixing, 3 mL of MTBE was added, and the mixture was vigorously shaken for 15 min. The sample was then centrifuged at 4000× *g* for 10 min to facilitate phase separation, after which the organic supernatant was carefully transferred. The extraction process was repeated with an additional 3 mL of MTBE, and the combined organic extracts were evaporated to complete dryness under nitrogen gas. The dried residue was reconstituted in 50 μL of methanol before analysis.

Quantification of 6:2 FTEOs in prepared extracts was performed using an ultrahigh-performance liquid chromatography (ACQUITY UPLC system) interfaced with a triple quadrupole mass spectrometer (Xevo TQ-S; Waters Co., Milford, MA, USA). Chromatographic separation was performed on a Accucore C18 column (2.6 μm particle size, 2.1 mm × 100 mm; Thermo-Scientific, Waltham, NY, USA) maintained at 40 °C. The mobile phase, delivered at 0.20 mL/min, consisted of two components—(A) pure water (containing 0.2% acetic acid and 5.0 mM ammonium acetate) and (B) a mixture of 50%/50% acetonitrile/isopropanol. The gradient elution program was set as follows: initial condition of 10% B (0–0.5 min), linear increase to 45% B at 1.0 min, followed by a gradient increase to 95% B (at 9.0 min, maintained for 2 min), and subsequent re-equilibration at 10% B (achieved within 0.1 min and held for 4.0 min). Mass spectrometric detection of 6:2 FTEOs was performed in positive electrospray ionization mode with multiple reaction monitoring (MRM). Detailed information regarding MRM transition pairs and instrument parameters is provided in the Supplementary Materials, Table S2.

### 2.4. Determination of Lipids in Human Serum

The serum concentrations of TC, TG, HDL, and LDL were measured immediately following serum separation (100 μL) from the collected human blood. These analyses were performed using an automated biochemical analyzer (Seamaty; Chengdu, China) at the hospital facility.

### 2.5. QA/QC

Rigorous quality control procedures were systematically applied during the human serum sample preparation and extraction processes. Pure water control blanks were analyzed in parallel with human serum samples to evaluate potential background contamination of 6:2 FTEOs. The analytical sequence incorporated methanol solvent blanks after each set of ten serum specimens, along with procedural blanks containing 1.0 mL of pure water that were processed identically to human serum samples in batches of ten samples. All chemical reagents were subjected to exhaustive preliminary examination to ensure no detectable levels of 6:2 FTEOs.

Determination of 6:2 FTEO levels in human serum was accomplished through an internal standard calibration approach [10]. Quantitative analysis of various 6:2 FTEO homologues was conducted using calibration curves of 6:2 FTEO8, in accordance with published methodology [10]. The calibration protocol involved establishing a six-point calibration curve in the human serum matrix spanning concentrations from 0.50 to 100 ng/mL for each 6:2 FTEO homologue. This calibration curve exhibited outstanding linear response characteristics, with R^2^ greater than 0.995 throughout the validated analytical range.

The limits of detection (LODs) were determined using a signal-to-noise threshold ratio of 3:1 [27], yielding detectable concentration limits between 0.011 ng/mL and 0.094 ng/mL for target 6:2 FTEOs. Method accuracy was verified through extraction recovery studies employing human serum matrix fortified at three concentration levels, achieving extraction recovery rates from 81% to 106% across all 6:2 FTEO homologues. Precision evaluation of the analyzing method involved triplicate measurements of human serum containing 10 ng/mL or 100 ng/mL of commercial 6:2 FTEO mixture. Results demonstrated that the intra-day relative standard deviations were less than 10%, and inter-day variability was below 20% across a seven-day assessment period. Method validation data, including LODs and extraction recovery rates for all 6:2 FTEO homologues, are available in the Supplementary Materials, Table S3.

### 2.6. Statistical Analysis

The normality distribution for human serum concentrations of 6:2 FTEO homologues, target lipid concentrations, and other continuous covariates was verified through the Shapiro–Wilk normality test. For 6:2 FTEOs with measured concentrations below LODs, non-detected values were imputed by dividing the LODs by the square root of two. Mean serum concentration calculations were restricted to 6:2 FTEOs demonstrating detection frequencies exceeding 50%. Relationships among serum levels of various 6:2 FTEOs were investigated using Spearman’s rank-order correlation coefficients. Comparative analyses of concentration disparities between different 6:2 FTEOs, as well as gender-specific serum concentrations of various 6:2 FTEOs, were conducted through the nonparametric Mann–Whitney *U* test.

In addition, a multiple linear regression (MLR) framework was implemented to systematically evaluate potential dose–response relationships between 6:2 FTEO exposure and five serum lipid parameters. To mitigate right-skewed distribution characteristics, all 6:2 FTEO concentration data underwent natural logarithmic transformation prior to inclusion in analytical models. This regression model incorporated demographic and physiological covariates, including age, sex, body mass index, educational level, occupational status, marital status, residence, alcohol intake, and tobacco smoke (as shown in Table 1), which were selected based on previously documented associations with lipid homeostasis [25,28,29]. Each target lipid (TC, TG, LDL-C, and HDL-C) was modeled as a continuous dependent variable. Exposure-effect estimates for serum lipid dysregulation were expressed as adjusted beta coefficients (*B*) with the corresponding 95% confidence intervals (CI). To validate the adjusted multivariate linear regression model, we conducted multicollinearity checks (all VIFs < 5) and dose–response verification using spline regression (*p* for nonlinearity > 0.10). All computational procedures were executed using the SAS statistical software suite (version 9.4, SAS Institute Inc., Cary, NC, USA), with statistical significance determined by a two-tailed probability threshold of 5%.

## 3. Results and Discussion

### 3.1. Demographic Characteristics of Study Subjects

The mean age of the males and females in this study were 40 ± 11 years and 41 ± 13 years, respectively, as shown in Table 1. Around 80% of study participants lived in the urban region of Hangzhou City. Most of the participants were employed (75% for males and 64% for females) and married (88% for males and 75% for females). Alcohol consumption (39% vs. 12%) and tobacco smoking (32% vs. 11%) were more prevalent in males than that in females.

### 3.2. Detection of 6:2 FTEOs in Human Serum

**Figure 1:**
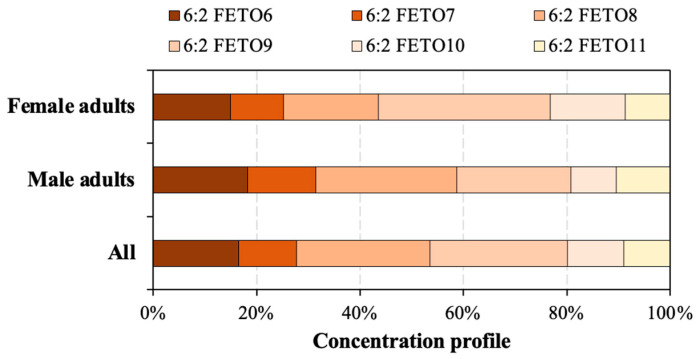
Concentration profiles of 6:2 FTEOs in human serum from male adults, female adults, and all participants.

Analyzing collected human serum showed that nine 6:2 FTEO homologues (i.e., 6:2 FTEO5–6:2 FTEO13) were detectable, with the detection frequencies of 22–81% (Table 2). The total human serum concentration of 6:2 FTEOs (∑6:2 FTEOs) was <LOD-17 ng/mL (mean 2.7 ng/mL). 6:2 FTEO6–6:2 FTEO9 were the more frequently detected 6:2 FTEO homologues, with the detection frequencies higher than 70%. This may suggest that the general Chinese population had a wide exposure to these 6:2 FTEO homologues. 6:2 FTEO8 and 6:2 FTEO9 were the more abundant 6:2 FTEO homologues in human serum, displaying the mean levels of 0.69 ng/mL (range < LOD–7.36 ng/mL) and 0.71 ng/mL (<LOD–8.12 ng/mL), respectively, which was followed by 6:2 FTEO6 (mean 0.44 ng/mL, range < LOD–3.03 ng/mL) and 6:2 FTEO7 (0.30 ng/mL, range < LOD–3.15 ng/mL). On average, 6:2 FTEO8 and 6:2 FTEO9 contributed 25% and 26% of ∑6:2 FTEOs, respectively (Figure 1). Given the high prevalence of these 6:2 FTEOs detected in human serum, there is growing concern about their ability to bioaccumulate in the human body.

Previous monitoring studies found that 6:2 FTEO8 and 6:2 FTEO9 were also among the predominant 6:2 FTEOs in antifog wipes, cloths, sprays, and indoor dust samples from USA and China [6,10]. This is consistent with this study. Cheng et al. reported that the 6:2 FTEO8−6:2 FTEO11 were the frequently detected 6:2 FTEOs in human serum, with the median levels of 0.13–0.22 ng/mL [10]. These reported levels are lower than that observed in this study. In addition, correlation analysis showed that human serum concentrations of 6:2 FTEO8, 6:2 FTEO9, and 6:2 FTEO10 were significantly correlated with one another (Spearman’s correlation coefficient, *r*_s_ = 0.47–0.83, *p* < 0.038) (Supplementary Materials, Table S4). This may indicate that participants were exposed to these 6:2 FTEOs through similar exposure sources, or that these 6:2 FTEOs had similar metabolic behaviors in the human body. Despite monitoring studies revealing the presence of varying 6:2 FTEOs in many environmental matrixes, sources of human exposure to these emerging PFASs are still unclear, which warrants more studies.

### 3.3. Gender- and Age-Specific Distinctions

**Figure 2:**
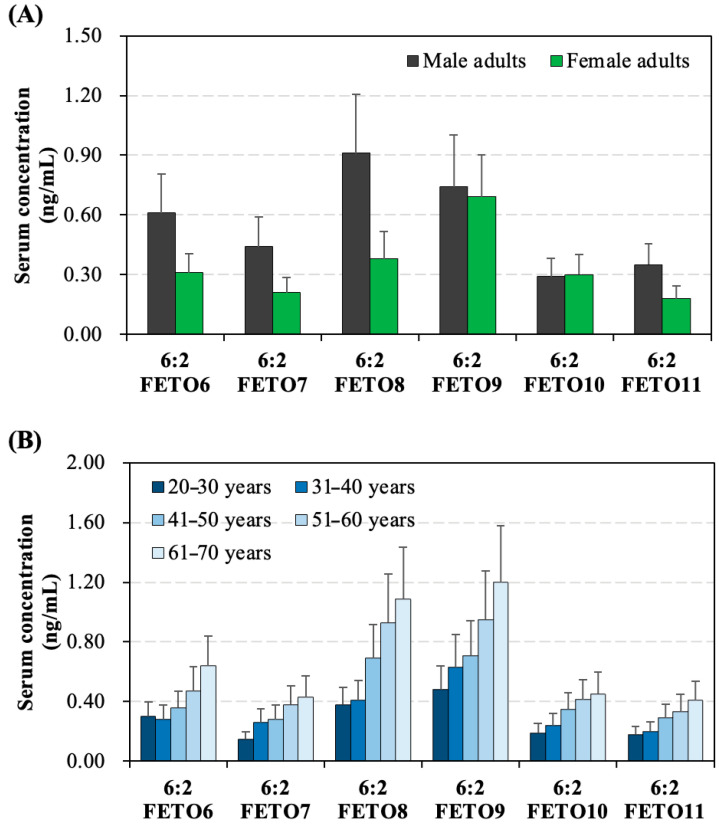
(**A**) Concentrations (mean ± SD) of 6:2FTEOs in human serum samples from male and female adults. (**B**) Concentrations (mean ± SD) of PPDs in human serum samples from participants in different age groups.

This biomonitoring study investigated gender-based differences in human serum concentrations of detected 6:2 FTEOs, as illustrated in Figure 2A. The comparison results showed that male subjects had much higher mean serum levels of 6:2 FTEO6, 6:2 FTEO7, 6:2 FTEO8, and 6:2 FTEO11 compared to female subjects. Mean level of 6:2 FTEO9 was also higher in serum samples from male subjects than that from female subjects, although this difference was not statistically significant (*p* > 0.05). These gender-specific disparities in serum levels of 6:2 FTEOs may be attributed to several reasons. One potential explanation is that male participants may have higher exposure to 6:2 FTEOs due to increased contact with products containing these compounds, such as antifog wipes, cloths, and sprays. Additionally, biological differences between sexes, including variations in hormone levels, body composition, kidney function, and enzyme activity, could affect how 6:2 FTEOs are metabolized and excreted in the human body [30,31]. For instance, women may eliminate more 6:2 FTEOs through unique pathways like breastfeeding and menstruation, a pattern also observed with other PFASs like PFOA and PFOS.

To assess the influence of human age on 6:2 FTEO levels in human serum, recruited participants were divided into four age groups—20–30 years (*n* = 55), 31–40 years (*n* = 78), 41–50 years (*n* = 70), 51–60 years (*n* = 35), and 61–70 years (*n* = 35). The average concentration of 6:2 FTEO6–6:2 FTEO11 in human serum showed a consistently steady increase trend from subjects aged 20–30 years to those who in the 51–61 years age group, as illustrated in Figure 2B. In addition, the correlation analysis also revealed a significantly positive relationship (*r*_s_ = 0.49–0.70, *p* < 0.01) between the age of participants and the human serum concentrations of 6:2 FTEO6–6:2 FTEO11. The observed age-related trend highlights the importance of considering age when evaluating human exposure to 6:2 FTEOs. This increase could be attributed to the declining kidney function in older individuals [32,33], impairing their ability to efficiently eliminate these compounds. Cheng et al. revealed that 6:2 FTEO7–6:2 FTEO12 bind to transport proteins (e.g., human serum albumin and L-FABP) with the affinity comparable to PFOA and PFOS [10]. This probably results in their prolonged circulation in human blood and long half-lives. Additional factors, such as dietary changes, reduced physical activity, hormonal fluctuations, and lower exposure to 6:2 FTEOs in older populations, may also contribute to this trend.

### 3.4. Associations Between 6:2 FETO Exposure and Dyslipidemia

**Figure 3:**
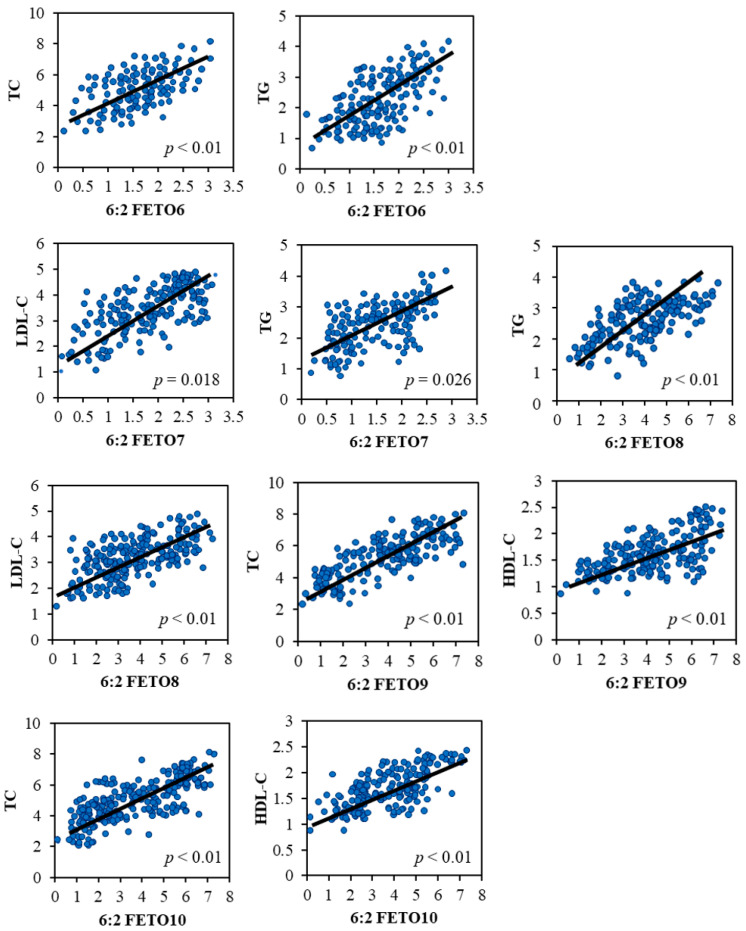
Significant correlations between serum 6:2 FETO levels and lipid levels in humans.

To explore the potential effect of human exposure to 6:2 FETOs on the lipid metabolism, we measured the human serum concentrations of TC, HDL-C, LDL-C, and TG (Table 3) and further investigated their relationships with serum concentrations of 6:2 FETOs (Figure 3). Table 4 displays the MLR coefficients between concentrations of 6:2 FETOs and various lipids in human serum samples. MLR analysis demonstrated that after adjustment for covariables, serum concentrations of TC were positively (statistically significant) associated with that of 6:2 FETO6 (*β* = 0.39, 95% CI 0.23–1.40, *p* < 0.01), 6:2 FETO9 (*β* = 0.44, 95% CI 0.084–2.08, *p* = 0.039), and 6:2 FETO10 (*β* = 0.62, 95% CI 1.03–3.11, *p* < 0.01). Human serum concentrations of HDL-C were positively (statistically significant) associated with that of 6:2 FETO9–6:2 FETO10 (*β* = 0.41–0.57, *p* < 0.020). Concentrations of LDL-C in human serum samples were positively (statistically significant) associated with those of 6:2 FETO7 (*β* = 0.55, 95% CI 1.04–2.29, *p* = 0.028) and 6:2 FETO8 (*β* = 0.68, 95% CI 1.21–4.25, *p* < 0.01). Concentrations of TG in human serum samples were positively (statistically significant) associated with those of 6:2 FETO6–6:2 FETO8 (*β* = 0.41–0.64, *p* < 0.040).

The biological mechanism leading to the associations between serum 6:2 FETO levels and lipid levels in humans is still largely unknown. Cheng et al. indicated that 6:2 FETOs may competitively interact with liver fatty acid-binding protein (L-FABP), leading to the subsequent activation of peroxisome proliferator-activated receptor alpha (PPAR-α) [10]. This activation influences the expression of proteins associated with lipid transport and biosynthesis, consequently disrupting lipid metabolism in the human body [34,35]. PPAR-α plays a fundamental role in fatty acid degradation and modulates the function of enzymes involved in lipid oxidation [36,37,38]. Recent in vitro data on murine 3T3-L1 cells also demonstrated that exposure to 6:2 FETOs induced substantial cytotoxic effects and promotes adipogenesis through enhanced TG deposition and stimulation of preadipocyte multiplication [6]. Furthermore, the direct molecular interaction between 6:2 FETOs and various human serum proteins may induce structural modifications in protein secondary conformation, while impairing their transport capabilities [39]. Such alterations could subsequently interfere with several essential physiological processes, such as endocrine regulation and immune system functionality [40,41]. Additionally, similar to PFOA and PFOS, 6:2 FETOs appear to negatively impact lipid homeostasis through the inhibition of the hepatic nuclear factor 4α receptor activity [42], which is a critical transcriptional controller of various lipid metabolic pathways [43].

While the affinity of PFAS for lipids has been well-documented, our study provides novel evidence that 6:2 FTEOs, a less-studied subclass of PFAS, disrupt lipid homeostasis in humans. Overall, the observed associations between specific 6:2 FTEO homologues (e.g., 6:2 FTEO6–6:2 FTEO10) and elevated serum lipids (e.g., TC, LDL-C, HDL-C, and TG) may arise through multiple pathways, including competitive binding to lipid transport proteins, PPAR-α activation, and hepatic nuclear receptor disruption. Unlike legacy PFAS, 6:2 FTEOs exhibit ethoxylate side chains that may enhance their solubility and bioavailability, potentially intensifying interactions with lipid membranes or receptors. This study bridges the gap between epidemiological observations and mechanistic plausibility, underscoring the need to evaluate newer PFAS subclasses separately from traditional compounds. Despite that, further investigation is required to fully elucidate the exact molecular pathways through which 6:2 FETO exposure influences the lipid metabolism in the human body.

The findings of this study highlight potential clinical significance in several aspects. First, the observed associations between 6:2 FTEO exposure and dyslipidemia (elevated TC, LDL-C, and TG) suggest that these emerging PFAS may contribute to cardiovascular risk factors, warranting consideration in routine lipid screening for populations with high exposure (e.g., occupational settings or anti-fog product users). Second, the gender- and age-specific disparities in serum 6:2 FTEO levels imply that tailored risk assessments and interventions may be needed, particularly for males and older adults who exhibited higher accumulation. Clinicians could integrate PFAS exposure history into diagnostic evaluations for unexplained lipid abnormalities.

### 3.5. Limitations of the Present Study

While this study provides valuable insights into the association between 6:2 FTEO exposure and serum lipid levels, several limitations should be acknowledged. First, the cross-sectional design precludes establishing causal relationships between 6:2 FTEOs and dyslipidemia. Second, the study population was limited to healthy adults from Hangzhou, China, which may restrict the generalizability of the findings to other regions or populations with different demographics or health statuses. Third, potential confounding factors, such as dietary habits and genetic predispositions, were not fully accounted for, which might influence lipid metabolism independently. Fourth, the detection of 6:2 FTEOs relied on serum concentrations, which may not fully reflect long-term exposure or cumulative effects. Lastly, the mechanisms underlying the observed associations remain speculative, necessitating further experimental and longitudinal studies to elucidate the biological pathways involved. Addressing these limitations in future research would strengthen the understanding of 6:2 FTEOs’ health impacts.

## 4. Conclusions

To our knowledge, no previous human studies have examined the health effects of 6:2 FTEOs. Therefore, our study represents the first investigation into the potential impact of 6:2 FTEO exposure on lipid profiles, contributing important preliminary evidence for future research in this field. Nine 6:2 FTEO homologues (i.e., 6:2 FTEO5–6:2 FTEO13) were detected in collected human serum, with the dominance of 6:2 FTEO8 and 6:2 FTEO9. More studies are necessary to elucidate the sources of human exposure to these 6:2 FTEO homologues. Male participants had much higher (*p* < 0.05) mean serum levels of 6:2 FTEO6, 6:2 FTEO7, 6:2 FTEO8, and 6:2 FTEO11 than female subjects. Gender-specific disparities in human 6:2 FETO exposure levels, coupled with age-dependent accumulation patterns of 6:2 FETOs in human serum, underscore the need for accurate risk assessments of these emerging contaminants. Statistical analysis revealed a significantly positive relationship between age of participants and the human serum concentrations of 6:2 FTEO6–6:2 FTEO11. For the first time, findings of this study revealed significant positive associations between some 6:2 FTEO homologues (e.g., 6:2 FTEO6 and 6:2FTEO8–6:2FTEO10) and elevated serum lipids (TC, LDL-C, HDL-C, and TG), suggesting their potential role in disrupting lipid homeostasis and increasing cardiovascular disease risks. Future research should prioritize longitudinal studies to assess long-term 6:2 FTEO exposure health impacts and to elucidate the potential interactions with lipid-regulatory pathways (e.g., PPAR-α and L-FABP).

## Figures and Tables

**Table 1:** Demographic characteristics of male and female participants recruited in this study.

	Males (*n* = 115)		Females (*n* = 122)	
	*n*	Percentage	*n*	Percentage
Age (Years)				
20–30	19	17%	18	15%
31–40	25	22%	31	25%
41–50	20	17%	19	16%
51–60	28	24%	32	26%
61–70	23	20%	22	18%
Body mass index (kg/m^2^)				
<18.5	24	21%	30	25%
18.5–24.5	60	52%	78	64%
>24.5	31	27%	14	11%
Educational level				
Lower than high school	35	30%	37	30%
High school	47	41%	39	32%
College	33	29%	46	38%
Occupational status				
Employed	86	75%	78	64%
Unemployed	29	25%	44	36%
Marital status				
Married	101	88%	92	75%
Unmarried	14	12%	30	25%
Residence				
Urban region	93	81%	98	80%
Rural region	22	19%	24	20%
Alcohol consumption				
No	70	61%	107	88%
Yes	45	39%	15	12%
Tobacco smoker				
No	78	68%	109	89%
Yes	37	32%	13	11%

**Table 2:** Concentration distribution (ng/mL) of 6:2 FTEOs in human serum (*n* = 237).

				Percentile		
	Detection Frequency	Mean	25th	50th	75th	Max
6:2 FETO2	0%	NC	<LOD	<LOD	<LOD	<LOD
6:2 FETO3	0%	NC	<LOD	<LOD	<LOD	<LOD
6:2 FETO4	0%	NC	<LOD	<LOD	<LOD	<LOD
6:2 FETO5	30%	NC	<LOD	<LOD	0.26	1.17
6:2 FETO6	71%	0.44	<LOD	0.43	1.16	3.03
6:2 FETO7	79%	0.30	0.10	0.41	1.17	3.15
6:2 FETO8	81%	0.69	0.41	0.45	4.02	7.36
6:2 FETO9	76%	0.71	0.098	0.80	4.11	8.12
6:2 FETO10	63%	0.29	<LOD	0.31	1.49	2.90
6:2 FETO11	63%	0.24	<LOD	0.17	1.10	4.35
6:2 FETO12	44%	NC	<LOD	<LOD	0.28	1.77
6:2 FETO13	22%	NC	<LOD	<LOD	<LOD	<LOD
6:2 FETO14	0%	NC	<LOD	<LOD	<LOD	<LOD
6:2 FETO15	0%	NC	<LOD	<LOD	<LOD	<LOD
6:2 FETO16	0%	NC	<LOD	<LOD	<LOD	<LOD
6:2 FETO17	0%	NC	<LOD	<LOD	<LOD	<LOD
6:2 FETO18	0%	NC	<LOD	<LOD	<LOD	<LOD

**Table 3:** Concentrations (mM) of TC, HDL-C, LDL-C, and TG in human serum.

	Detection Frequency	Mean	Median	Range
TC	100%	5.26	5.39	2.30–8.07
HDL-C	100%	1.68	1.78	0.89–2.45
LDL-C	100%	2.72	3.01	1.05–4.77
TG	100%	2.25	2.60	0.62–4.14

**Table 4:** Associations in serum concentrations between 6:2 FTEOs and lipid revealed by the multivariate linear regression analysis.

	Beta, (95% CI), *p*-Value			
	TC	HDL-C	LDL-C	TG
6:2 FETO6	0.81 ** (0.23–1.40)	−0.21 (−0.43–0.05)	0.082 (−0.29–0.28)	0.99 * (0.49–1.47)
6:2 FETO7	2.42 (−1.6–6.09)	3.29 (−2.14–5.56)	1.16 * (1.04–2.29)	2.13 * (1.01–3.46)
6:2 FETO8	−1.27 (−2.79–0.33)	−2.23 (−3.53–0.99)	3.17 ** (1.21–4.25)	1.95 ** (4.26–4.24)
6:2 FETO9	0.94 * (0.084–2.08)	2.46 ** (1.01–3.79)	0.45 (−2.80–3.51)	−0.53 (3.71–4.27)
6:2 FETO10	2.07 ** (1.03–3.11)	1.01 * (0.34–1.73)	−0.79 (−1.46–0.13)	−2.06 (−2.44–0.38)
6:2 FETO11	0.63 (−4.20–5.46)	0.77 (−1.45–3.03)	0.12 (−0.47–0.66)	0.089 (−1.38–1.43)

Note that * means *p* < 0.05 and ** means *p* < 0.01.

## Data Availability

The datasets generated and/or analyzed during the current study are available from the corresponding author on reasonable request. Due to privacy restrictions related to human sample data, the raw data are not publicly available.

## Supplementary Materials

The following supporting information can be downloaded at https://www.mdpi.com/article/10.3390/toxics13080664/s1, Figure S1: Chemical structures of 6:2 FTEO homologues in this study; Table S1: Accurate content of each 6:2 FTEO homologue in the commercial 6:2 FTEO mixture (trade name FS-3100); Table S2: MRM parameters for detecting 6:2 FTEOs and internal standards; Table S3: LODs and extraction recovery of 6:2 FTEOs in human serum; Table S4: Correlations among concentrations of 6:2 FTEOs in human serum samples.
