# Supplementary material for: Association of 6:2 Fluorotelomer Ethoxylate Exposure with Serum Lipids in General Adults
Source: Toxics. 2025 Aug 7;13(8):664. doi: 10.3390/toxics13080664 (PMC12389903; doi:10.3390/toxics13080664)
Supplement: Supplementary file 1 [file toxics-13-00664-s001.zip › toxics-3693181-supplementary.pdf]

SUPPORTING INFORMATION

Association of 6:2 Fluorotelomer Ethoxylate Exposure with Serum Lipids in General Adults

Yan Wu[1,2]; Qianjin Li[2]; Rendi Deng[2]; Rui Wang[2]; Junfen Fu[1*]; Fangfang Ren[3]; Hangbiao Jin[3]

[1] Department of Endocrinology, Children's Hospital, Zhejiang University School of Medicine, National Clinical Research Center for Child Health, Hangzhou, Zhejiang 310014, P. R. China.

[2] Greentown Cardiovascular Disease Hospital, Zhejiang University, Hangzhou, Zhejiang 310014, P. R. China.

[3] Key Laboratory of Microbial Technology for Industrial Pollution Control of Zhejiang Province, College of Environment, Zhejiang University of Technology, Hangzhou, Zhejiang 310014, P. R. China.

Table S1. Accurate Content of Each 6:2 FTEO Homologue in the Commercial 6:2 FTEO Mixture (Trade Name FS-3100).

	Content
**6:2FTE2**	5.0%
**6:2FTE3**	5.0%
**6:2FTE4**	5.0%
**6:2FTE5**	5.0%
**6:2FTE6**	5.0%
**6:2FTE7**	5.0%
**6:2FTE8**	10%
**6:2FTE9**	5.0%
**6:2FTE10**	10%
**6:2FTE11**	5.0%
**6:2FTE12**	10%
**6:2FTE13**	5.0%
**6:2FTE14**	5.0%
**6:2FTE15**	5.0%
**6:2FTE16**	5.0%
**6:2FTE17**	5.0%
**6:2FTE18**	5.0%

Table S2. MRM Parameters for Detecting 6:2 FTEOs and Internal Standards.

	Parent ion (*m/z*)	Cone voltage (eV)	Daughter ion (*m/z*)	Collision energy (eV)
**6:2FTE2**	470.1	45	391	19
	470.1	45	89	25
**6:2FTE3**	514.1	45	391	23
	514.1	50	89	30
**6:2FTE4**	558.2	45	391	16
	558.2	50	89	28
**6:2FTE5**	602.2	45	391	20
	602.2	50	89	37
**6:2FTE6**	646.2	50	391	18
	646.2	50	89	33
**6:2FTE7**	690.2	50	391	27
	690.2	45	89	35
**6:2FTE8**	734.3	45	391	27
	734.3	50	89	34
**6:2FTE9**	778.3	50	391	26
	778.3	45	89	40
**6:2FTE10**	822.3	45	391	20
	822.3	50	89	30
**6:2FTE11**	866.3	45	391	22
	866.3	45	89	30
**6:2FTE12**	910.4	50	391	22
	910.4	45	89	29
**6:2FTE13**	954.4	50	391	20
	954.4	50	89	37
**6:2FTE14**	998.4	50	391	18
	998.4	50	89	33
**6:2FTE15**	1042.4	45	391	29
	1042.4	45	89	35
**6:2FTE16**	1086.5	50	391	27
	1086.5	50	89	45
**6:2FTE17**	1130.5	45	391	23
	1130.5	45	89	48
**6:2FTE18**	1174.5	50	391	34
	1174.5	50	89	43
**Benzyldimethyldodecylammonium chloride-d_5**	309	40	96	24
**Benzyldimethyltetradecylammonium chloride-d_7**	339	40	98	32

Table S3. LODs and Extraction Recovery of 6:2 FTEOs in Human Serum.

	LOD (ng/mL)	Extraction recovery (%) Spiked at 10 ng/mL of commercial 6:2 FTEO mixture		Spiked at 50 ng/mL of commercial 6:2 FTEO mixture		Spiked at 250 ng/mL of commercial 6:2 FTEO mixture	
		Mean	SD	Mean	SD	Mean	SD
**6:2 FTEO2**	0.031	106	8	94	14	83	8
**6:2 FTEO3**	0.049	92	5	103	12	97	12
**6:2 FTEO4**	0.062	91	15	90	14	104	7
**6:2 FTEO5**	0.019	104	14	99	10	88	9
**6:2 FTEO6**	0.066	98	7	90	13	89	11
**6:2 FTEO7**	0.071	88	9	85	10	84	6
**6:2 FTEO8**	0.052	94	8	87	5	93	5
**6:2 FTEO9**	0.011	98	4	86	14	101	11
**6:2 FTEO10**	0.060	84	12	83	5	99	15
**6:2 FTEO11**	0.048	105	9	84	8	87	15
**6:2 FTEO12**	0.094	99	6	100	12	86	11
**6:2 FTEO13**	0.022	86	12	90	9	83	13
**6:2 FTEO14**	0.080	84	9	95	14	109	7
**6:2 FTEO15**	0.072	97	13	81	14	101	6
**6:2 FTEO16**	0.076	103	7	106	7	82	15
**6:2 FTEO17**	0.031	87	6	90	15	89	12
**6:2 FTEO18**	0.081	86	8	82	12	103	8

Table S4. Correlations among Concentrations of 6:2 FTEOs in Human Serum Samples.

	6:2 FETO5	6:2 FETO6	6:2 FETO7	6:2 FETO8	6:2 FETO9	6:2 FETO10	6:2 FETO11	6:2 FETO12
**6:2 FETO6**	$r_s = 0.11$<br>$p = 0.21$	1						
**6:2 FETO7**	$r_s = 0.39$<br>$p = 0.09$	$r_s = 0.15$<br>$p = 0.30$	1					
**6:2 FETO8**	$r_s = 0.11$<br>$p = 0.49$	$r_s = 0.76$<br>$p < 0.01$	$r_s = 0.58$<br>$p < 0.01$	1				
**6:2 FETO9**	$r_s = 0.62$<br>$p < 0.01$	$r_s = 0.61$<br>$p < 0.01$	$r_s = 0.32$<br>$p = 0.15$	$r_s = 0.78$<br>$p < 0.01$	1			
**6:2 FETO10**	$r_s = 0.49$<br>$p = 0.038$	$r_s = 0.83$<br>$p < 0.01$	$r_s = 0.79$<br>$p < 0.01$	$r_s = 0.52$<br>$p < 0.01$	$r_s = 0.58$<br>$p = 0.02$	1		
**6:2 FETO11**	$r_s = 0.12$<br>$p = 0.44$	$r_s = 0.58$<br>$p < 0.01$	$r_s = 0.59$<br>$p < 0.01$	$r_s = 0.47$<br>$p < 0.01$	$r_s = 0.13$<br>$p = 0.49$	$r_s = 0.60$<br>$p < 0.01$	1	
**6:2 FETO12**	$r_s = 0.56$<br>$p < 0.01$	$r_s = 0.19$<br>$p = 0.34$	$r_s = 0.17$<br>$p = 0.47$	$r_s = 0.46$<br>$p = 0.09$	$r_s = 0.34$<br>$p = 0.19$	$r_s = 0.26$<br>$p = 0.19$	$r_s = 0.36$<br>$p = 0.14$	1
**6:2 FETO13**	$r_s = 0.43$<br>$p = 0.07$	$r_s = 0.25$<br>$p = 0.27$	$r_s = 0.31$<br>$p = 0.19$	$r_s = 0.73$<br>$p < 0.01$	$r_s = 0.45$<br>$p = 0.09$	$r_s = 0.31$<br>$p = 0.11$	$r_s = 0.17$<br>$p = 0.51$	$r_s = 0.36$<br>$p = 0.12$

Note that r_s means Spearman's correlation coefficient.

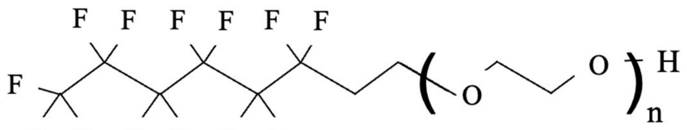

n = 2–18

6:2 fluorotelomer ethoxylates (6:2 FTEOs)

Figure S1. Chemical structures of 6:2 FTEO homologues in this study.
